# Immunization protected well nourished mice but not undernourished ones from lung injury in Methicillin-resistant *Staphylococcus aureus* (MRSA) infection

**DOI:** 10.1186/1471-2180-9-240

**Published:** 2009-11-23

**Authors:** Thais Graziela Donegá França, Larissa Lumi Watanabe Ishikawa, Sofia Fernanda Gonçalves Zorzella-Pezavento, Fernanda Chiuso-Minicucci, Clara Pires Fujiara Guerino, Maria de Lourdes Ribeiro de Souza da Cunha, Alexandrina Sartori

**Affiliations:** 1 Department of Microbiology and Immunology, Institute of Biosciencies, São Paulo State University (UNESP), Botucatu, Brazil, Distrito de Rubião Junior s/n, CEP: 18.618-000

## Abstract

**Background:**

*Staphylococcus aureus *methicillin-resistant (MRSA) has been frequently isolated from endotracheal and lung puncture aspirates in malnourished children with pneumonia. In this work we evaluated the susceptibility of undernourished BALB/c mice and its ability to mount a protective immunity against MRSA with emphasis on the lung involvement.

**Results:**

BALB/c mice submitted to a 20% dietary restriction during 20 days presented a significant decrease in body weight, lymphocyte number and also atrophy in thymus and intestinal epithelium. Determination of bacterial load by the number of colony forming units (CFU) indicated a similar susceptibility whereas the findings of Gram stain clearly suggested a higher amount of bacteria in the lungs of normal mice than in the undernourished ones. Immunization reduced bacterial growth in the lungs of normal mice but not in the undernourished ones. Histopathological analysis showed that inflammation appeared in the lungs from normal mice only after infection and that immunization prevented this pulmonary inflammatory process. On the other hand, undernourished mice presented lung inflammation even before infection. In addition, the degree of this inflammatory process did not change with infection or previous immunization.

**Conclusion:**

Our results indicated that lung injury during MRSA infection is prevented by previous immunization in well nourished but not in undernourished mice.

## Background

Protein energy malnutrition (PEM) is the most frequent type of malnutrition, affecting at least 800 million people worldwide [1]. It is especially prevalent in certain groups as children, elderly people, patients with chronic diseases or neoplasia, and also in 50 to 90% of hospitalized patients [2,3]. Malnutrition by itself can cause death [4] but epidemiological data reveals that it greatly increases susceptibility to and severity of infections, being a major cause of illness and death from infectious diseases [3,5]. A direct correlation between higher degrees of malnutrition and higher risk of death is supported by the observation that severely malnourished children experienced substantially higher mortality rates [6,7]. Increased morbidity and mortality in malnutrition is associated with decreased immunocompetence with particular involvement of cell-mediated immunity, antibody secretion and affinity and also complement components and cytokine production [8]. We recently demonstrated that diet restriction reduced IL-4 and IFN-γ and also abrogated specific antibody production in BALB/c mice immunized with a genetic vaccine containing the mycobacterial hsp65 gene [9]. As described above, a significant proportion of hospitalized patients are undernourished and at a greater danger to get severe hospital-infections. *Staphylococcus aureus *has been one of the most common bacterial causes of severe pneumonia in children with nosocomial infections [10]. Although previously considered as a purely nosocomial event, community-acquired methicillin-resistant *S. aureus *(MRSA) pneumonia is underestimated and is spreading worldwide [11]. In addition, leukocytopenia and malnutrition are described as high risk factors that lead to death by nosocomial *S. aureus *pulmonary infections [12]. In spite of its relevance, the behaviour of *S. aureus *in undernourished subjects has not been fully investigated.

In this context, we used a PEM murine model to evaluate both, the susceptibility and the ability to mount a protective immunity against a MRSA with emphasis on lung involvement.

## Results

### Alterations determined by undernutrition

**Figure 1:**
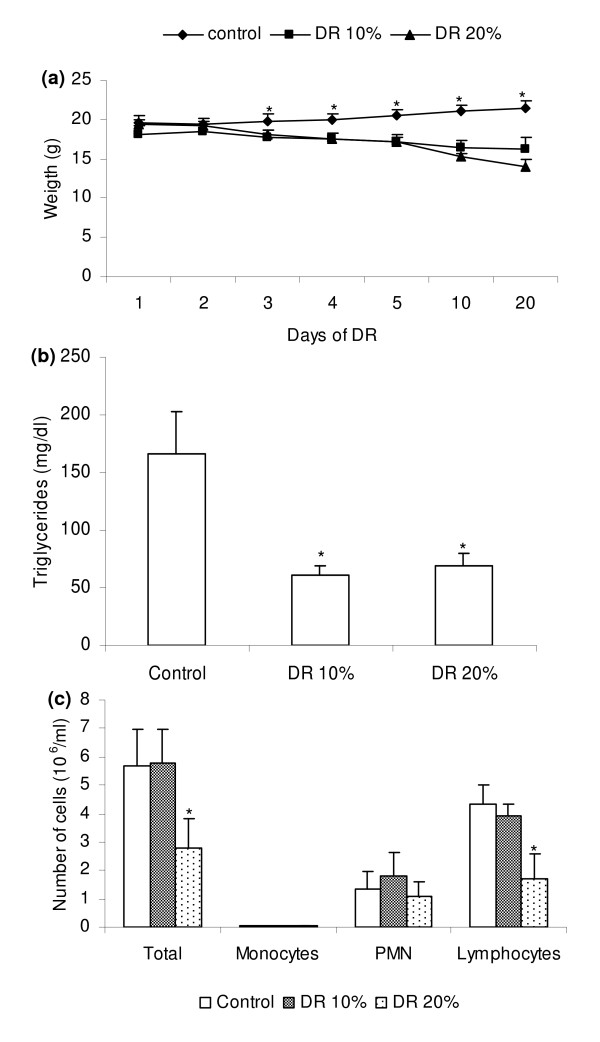
**Alterations determined by undernutrition**. BALB/c mice were submitted to two percentages of dietary restriction (10 and 20%) and evaluated in relation to weight loss (a), seric triglyceride concentration (b) and differential blood cell count (c). Results are expressed as mean ± SD of 5 animals per group (*p < 0.05) in relation to well nourished group.

We initially characterized a model of dietary restriction by determining body weight, triglyceride seric levels and leucogram. Effects of two percentages (10 and 20%) of dietary restriction were compared with parameters observed in a control group that received food *ad libitum*. Both levels of restriction determined a significant weight loss and decreased serum concentration of triglycerides (figure 1a and 1b, respectively). However, only the group submitted to 20% of dietary restriction presented alterations compatible with secondary immunodeficiency as decreased lymphocyte number (figure 1c).

### Effect of dietary restriction and immunization on bacterial load

**Figure 2:**
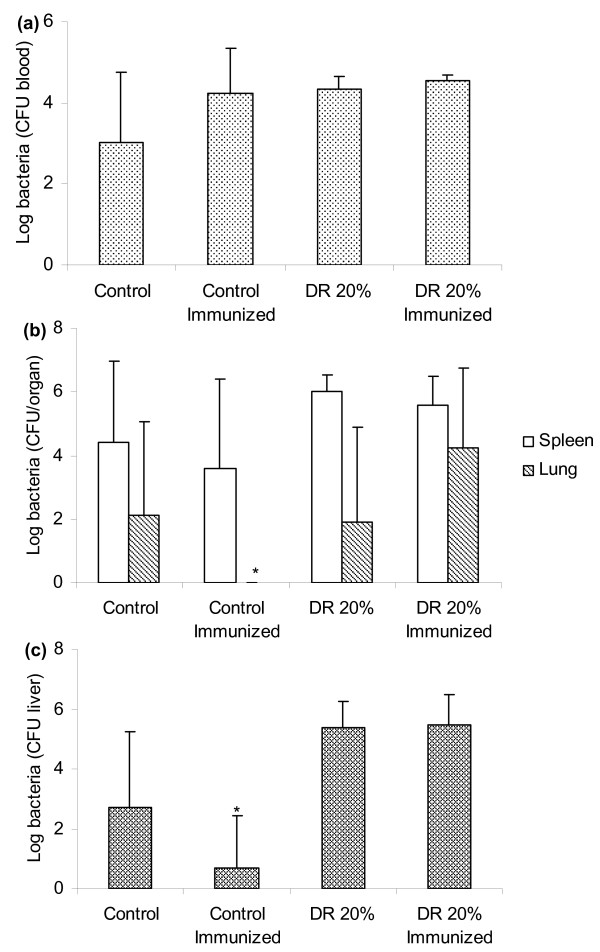
**Effect of dietary restriction and immunization on bacterial load**. BALB/c mice were submitted to dietary restriction (20%), immunized with the formolized bacteria and infected with 5 × 10^8 ^CFU/0.5 ml of *S. aureus*. The bacterial load was determined 24 hours later in the blood (a), spleen and lung (b) and liver (c). Results are expressed as mean ± SD of 5 animals per group (*p < 0.05) in relation to well nourished group.

Twenty-four hours after intraperitoneal infection with 5 × 10^8 ^CFU/0.5 mL of *S. aureus*, all animals from the four experimental groups presented bacteria in the blood (figure 2a). Determination of CFU in the spleen did not show any significant difference among these groups (figure 2b). However, differences were observed in lung analysis. Well nourished mice immunized with formolized *S. aureus *presented a significant reduction in CFU in this organ. Interestingly, this effect was not triggered in undernourished mice. An even increased amount of bacteria was present in undernourished immunized animals (figure 2b). A reduced amount of bacteria was also observed in the liver of well nourished mice that were previously immunized with *S. aureus *(figure 2c). Injection of Complete Freund's Adjuvant alone did not reduce bacterial load (not shown).

### Lung histopathological analysis

**Figure 3:**
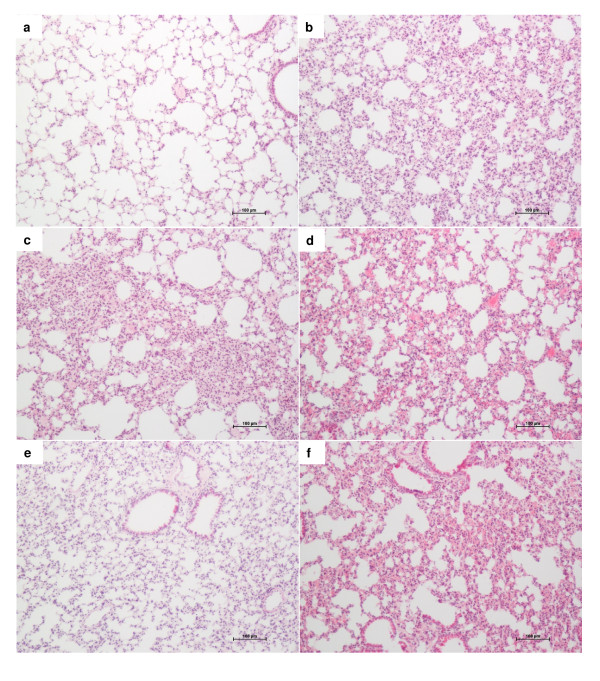
**Effect of dietary restriction and immunization on lung histology**. BALB/c mice were submitted to dietary restriction (20%), immunized with the formolized bacteria and infected with *S. aureus *(5 × 10^8 ^CFU/0.5 ml). Lung sections were obtained 24 hours later, stained with H&E and analysed with a Leica microscope. Lung samples from normal (a), undernourished (b), well nourished and infected (c), undernourished and infected (d), well nourished immunized and infected (e), undernourished immunized and infected (f).

As expected the pulmonary parenchyma from well nourished and non infected mice showed a very well preserved alveolar structure without any inflammatory process (figure 3a). Infection of well nourished animals determined a clear inflammatory infiltration in the lungs (figure 3c). This inflammatory reaction clearly subsided if the animals were immunized before infection (figure 3e). However, undernourished mice presented a distinct lung involvement. They already presented a pulmonary disseminated inflammatory process before infection with *S. aureus*. This reaction was characterized by septal thickening and a clear mononuclear cell infiltration (figure 3b). Interestingly, the intensity and the quality of this inflammatory reaction were not altered by infection preceded or not by immunization with killed *S. aureus*, as documented at figure 3d and 3f, respectively.

### Bacterial density evaluated by Gram stain

**Figure 4:**
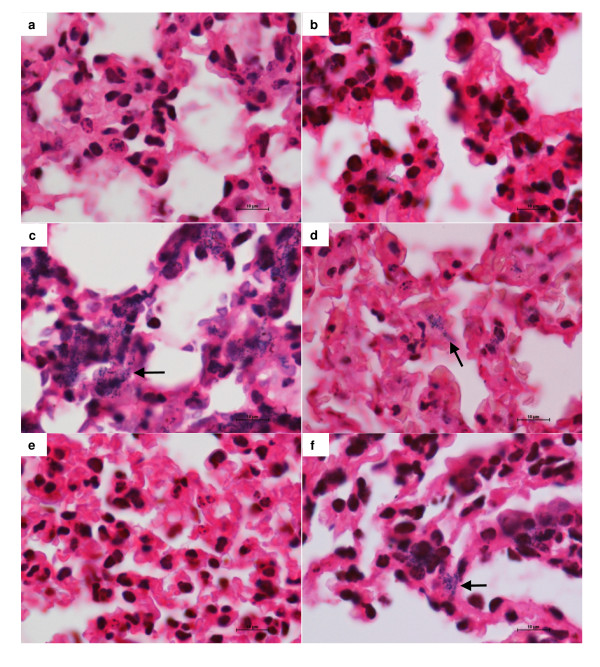
**Effect of dietary restriction and immunization on lung bacterial load**. BALB/c mice were submitted to dietary restriction (20%), immunized with the formolized bacteria and infected with *S. aureus *(5 × 10^8 ^CFU/0.5 ml). Lung sections were obtained 24 hours later, stained with Gram and analysed with a Nikon microscope. Lung samples from normal (a), undernourished (b), well nourished and infected (c), undernourished and infected (d), well nourished immunized and infected (e), undernourished immunized and infected (f). Arrows indicate bacteria location.

Staining of lung sections by Gram showed absence of the typical Gram positive cocci in non infected mice (figure 4a and 4b), independently of their nutritional status. A great amount of cocci was, as expected, present in infected well nourished mice (figure 4c). Immunization of these animals before infection visibly reduced the amount of these bacteria in lung parenchyma (figure 4e). Lung evaluation in undernourished mice indicated two striking differences. Comparing to well nourished group, the undernourished one presented a clear reduction in the amount of cocci in the lungs (figure 4d). In addition, previous immunization of these animals did not reduce lung colonization by the bacteria (figure 4f).

## Discussion

Protein energy malnutrition (PEM) is the most common type of undernutrition. It leads to secondary immunodeficiency and consequently increased susceptibility to infectious agents, including to *S. aureus *[13-15]. In this context, this work was done to establish a murine experimental model of PEM and to evaluate the effect of malnutrition on both, susceptibility and ability to mount a protective immunity against a methicillin-resistant *S. aureus *(MRSA). To define a model of dietary restriction, BALB/c female mice were submitted to a reduction of 10 or 20% in their food intake during 20 days and compared to a well nourished experimental group. Both levels of restriction determined a significant decrease in weight and serum triglycerides concentration. However, immunological evaluation indicated that only the group submitted to 20% dietary restriction developed secondary immunodeficiency. Initial comparison of colony forming units (CFU) obtained from spleen, liver and lung homogenates suggested that well nourished and undernourished mice were similarly susceptible to *S. aureus *infection. This methodology also suggested that a previous immunization with formolized *S. aureus *was able to partially protect healthy animals but not undernourished ones. In addition, this vaccine protective effect varied according to the evaluated organ; it was observed in the liver and lungs but not at the spleen. Even though determination of CFU in organs not previously perfused have been used as a parameter to quantify bacterial colonization [16] it is possible that bacteremia could interfere with the results. As lungs are critical targets during MRSA infections, a more detailed investigation was performed at the lungs by doing an histopathological analysis with H&E and Gram stains. This approach would allow a direct evaluation of lung parenchyma, avoiding a possible interference by bacteria present in the blood. As expected, lung structure was totally preserved among the animals from the normal control group that presented very well defined alveolar spaces and no signs of inflammation. Well nourished mice infected with *S. aureus *developed a clear and widespread inflammatory reaction in this organ. Interestingly, there was an evident downmodulation of this inflammatory reaction in well nourished mice previously vaccinated with *S. aureus*. On the other hand, undernourished animals already presented a lung disseminated inflammatory process before infection. This inflammatory reaction did not change in amount or quality after infection with *S. aureus *preceded or not by immunization. The cause of this inflammatory process was not investigated. However, it could be due to the presence of environmental agents or, alternatively, to the overgrow of resident bacteria that could trigger a respiratory infection in these animals but not in the well nourished ones.

As expected, staining of lung sections with Gram revealed a great amount of cocci in well nourished mice infected with *S. aureus*. Immunization before infection determined a visible reduction in the amount of bacteria and this coincided with an almost complete resolution of the inflammatory process found at the lung parenchyma. Comparing to these findings, two striking differences were detected in undernourished animals. They presented a much smaller amount of cocci in the lungs. This was initially unexpected because undernutrition has been more commonly associated with increased susceptibility to infectious agents [17]. However, this finding could be explained by competition for nutrients between host and pathogens as described by Prentice & McDermid, 2008 [18]; therefore decreasing the food supply for bacterial growth. Alternatively, endogenous or environmental bacteria could, as we said before, be already present at the pulmonary parenchyma in undernourished mice, competing for nutrients. The fact that *S. aureus *is a poor competitor and does not grow well in the presence of other microorganisms supports this hypothesis [19]. Previous immunization of undernourished mice, differently from the findings in the well nourished group, did not decrease the amount of cocci in the lungs. We believe that this result could be attributed, at least partially, to a decreased antibody production because they are essential to control *S. aureus *infections, including life-threatening conditions as pneumonia and septicemia [20].

From a practical point of view, these results raise two very relevant aspects. The first one relates to the condition of malnutrition as a high risk factor for nosocomial pulmonary infections caused by MRSA. This possibility has not been directly investigated but it has been suggested by some findings as the ones described by Miyake et al., 2007 [21]. Our results also alert for a possible low efficacy of an MRSA vaccine in undernourished patients, mainly concerning the prevention of pulmonary involvement.

## Conclusion

Together these results demonstrated that a 20% dietary restriction in food intake triggered a secondary immunodeficiency in BALB/c mice. This condition determined a very distinctive lung involvement in comparison to well nourished animals. This organ presented an inflammatory process that was not altered by infection with *S. aureus *or by infection preceded by immunization with the formolized bacteria. Absence of required nutrients or a state of resistance by the previous inflammatory process could decrease *S. aureus *growth in lungs of undernourished animals.

## Methods

### Experimental design

Isogenic female BALB/c mice, 4-5 weeks old were manipulated according to the ethical guidelines adopted by the Brazilian College of Animal Experimentation, being the experimental protocol approved by the local Ethics Committee. After weaning the animals received a 10 day acclimation on a standard chow. In the first set of experiments, after being acclimated they were distributed into three experimental groups (with 5-6 animals each) including the control fed *ad libitum *and two others that received 80 or 90% of the amount of food consumed by the control group and that were called DR 20% and DR 10%, respectively. The animals were kept in these conditions during 20 days and then evaluated by clinical (weight), biochemical (triglycerides) and lymphocyte number. In a second set of experiments, after being acclimated, mice were allocated into 4 experimental groups (4-5 animals each). Two groups were kept under normal diet and the other two were submitted to DR 20%. After 10 days, one control and one DR 20% group were immunized with formolized *S. aureus*. Ten days after, i.e, at the 20^th ^day from the beginning of diet, all groups were infected with a fresh *S. aureus *suspension. Twenty four hours later the animals were euthanized to determine the bacterial load by CFU in blood, spleen, liver and lungs. Lung injury was additionally evaluated by hematoxylin & eosin and Gram stains.

### Bacterial suspension

A *S. aureus *strain (S-6055/94) initially isolated from a clinical specimen was used for infections. This strain was characterized as being methicillin resistant by mecA gene detection by PCR. The strain was cultivated in blood agar and incubated at 37°C for 24 h. Isolated colonies were inoculated into brain heart broth and incubated at 37°C for 24 h. Bacteria were collected by centrifugation, washed and resuspended at a concentration of 1 × 10^9 ^CFU/mL. Mice were injected by intraperitoneal route with 5 × 10^8 ^CFU in 0.5 mL of saline. Control mice received an equal volume of saline. Bacteria were alternatively inactivated by resuspension in formol 3%. Normal and diet restricted groups (10^th ^day of restriction) were immunized by subcutaneous route with 2 × 10^8 ^CFU/0.2 ml formolized *S. aureus *previously emulsified with Complete Freund's Adjuvant.

### Blood evaluations

Blood samples were collected by cardiac puncture and total leukocyte number was counted after blood dilution in Turk's solution. Differential leukocyte count was performed by analysis of blood smears stained with eosin/methylene blue (Leishman's stain). Serum samples were kept al - 20°C and total triglycerides concentration was measured by an enzymatic method (Kits Laborlab, Guarulhos, São Paulo).

### Histopathological analysis

Lung sections were obtained 24 hours after infection, were fixed in formalin (10%), embedded in Paraplast plus (McCormick), prepared routinely and then sectioned for light microscopy. Sections (5 μm each) were stained with haematoxylin and eosin (H&E) or with Gram and analyzed by optical microscope and the images acquired with a coupled digital camera.

### Determination of blood and tissue bacterial loads

Blood samples, spleens, lungs and livers from infected animals were homogenized in saline and plated. Briefly, 0,1 mL of serially diluted organ homogenates or 50-100 μL of blood were inoculated into baird-parker agar plates and incubated at 37°C. Colonies were counted 24 h later.

### Statistical analysis

Statistical analysis was performed using SigmaStat statistical software (Jandel Corp., San Rafael, CA). The Kruskal-Wallis nonparametric test was used to compare CFU determinations in livers. For the parametric variables the results were expressed as mean ± standard deviation (SD) and the comparisons between the groups were made by variance analysis (ANOVA) followed by Tukey's test. A *P *value of less than 0.05% was considered statistically significant.

## Abbreviations

PEM: protein energy malnutrition; CFU: colony forming unit; H&E: haematoxylin and eosin; SD: standard deviation.

## Competing interests

The authors declare that they have no competing interests.

## Authors' contributions

TGDF and LLWI executed most of this work. SFGZP, FCM and CPFG. largely contributed with the immunological experiments and the statistical analysis. MLRSC. participated in the design of the study and contributed with her expertise in *Staphylococcus *and AS conceived the study, coordinated it and revised the manuscript. All authors read and approved the final manuscript.
